# Editorial: Genetic advances and translational applications in movement disorders

**DOI:** 10.3389/fneur.2023.1280958

**Published:** 2023-09-20

**Authors:** Giulietta M. Riboldi, Alessio B. Di Fonzo, Christopher D. Stephen

**Affiliations:** ^1^Department of Neurology, The Marlene and Paolo Fresco Institute for Parkinson's and Movement Disorders, NYU Langone Health, New York, NY, United States; ^2^Neurology Unit, Foundation IRCCS Ca' Granda Ospedale Maggiore Policlinico, Milan, Italy; ^3^Department of Neurology, Massachusetts General Hospital and Harvard Medical School, Boston, MA, United States

**Keywords:** genetics, movement disorders, counseling, whole exome sequencing, clinical trials

Despite recent advances in the field of genetics and translational medicine, many movement disorders still remain undiagnosed and difficult to treat. Genetic and genomic advances have led to the discovery of new genes and molecular pathways in movement disorders. Our Research Topic encompasses original works and literature reviews regarding the importance and challenges of genetic diagnosis, the pitfalls of atypical phenotypes, the benefit of leveraging interdisciplinary teams, and the potential for pathogenesis-directed therapies.

There are ongoing difficulties with clinic-genetic correlations in movement disorders (1, 2). Cesaroni et al. illustrate the importance of defining a genetic diagnosis with atypical symptoms. They describe a patient with a rare intellectual disability syndrome related to a pathogenic variant in *FOXP1*. Together with the classical phenotype, they describe paroxysmal dystonia and discuss the potential pathogenic role of *FOXP1* in the basal ganglia, which may lead to movement disorders in this population.

In addition, Ando et al. describe the prevalence of *RFC1* mutation carriers in a large cohort of patients with ataxia, who had a younger age of onset than previously described, in comparison to more typical late-onset presentations (3). As the causative role of intronic variants have been increasingly recognized as potentially common causes of genetic movement disorders, the inclusion of relevant intronic variants on gene panels and the assessment of repeat expansions are important to improve diagnosis (4).

More comprehensive tests, such as WES, are becoming more routine in the field of movement disorder as well. Zou et al. showed how WES allowed the identification of a new pathogenic variant in the *PLA2G6* gene in two siblings allowing immediate management of the affected probands and counseling for the family.

Genetic counseling is a key principle of the management of rare neurogenetic disorders and this is particularly important in the fatal prion diseases. Appleby et al. in their review discuss the genetic associations in sporadic, acquired, and genetic prion diseases and present the intricacies of genetic testing during life or at autopsy, in addition to the thorny ethical quandaries for testing of at-risk family members. They discuss the possibility of future treatments targeting the *PRNP* gene.

Given the complexity of rare genetic disorders, there is increasing use of an interdisciplinary team-based approach, such as the Undiagnosed Disease Program (5, 6). In their paper, Huang et al. describe the important experience of the White Matter Rounds network, an international, collaborative platform where clinicians and scientists discuss challenging cases, promoting new understanding of these complex diseases and leveraging different expertise to aid in the diagnosis and facilitate research in white matter disorders leukodystrophies.

There are understandable challenges in the design and implementation of clinical trials in rare neurological disorders. Videnovic et al. highlight the lessons learned from clinical trials in pantothenate kinase-associated neurodegeneration, discussing successful strategies that can be applied to other rare and particularly metabolic movement disorders. They suggest multi-disciplinary engagement with experts and approaches concerning selection and recruitment of appropriate patients, trial endpoints and duration, special statistical considerations, and the importance of regulatory feedback.

Therefore, as genetic diagnosis become more relevant in the landscape of movement disorders, diagnostic, and therapeutic approaches need to be reshaped to include new discoveries in clinical practice and maximize implementation of integrative resources from the clinical and research settings.

## Author contributions

GMR: Conceptualization, Writing—original draft, Writing—review and editing. ABD: Conceptualization, Writing—original draft, Writing—review and editing. CDS: Conceptualization, Writing—original draft, Writing—review and editing.
